# Workload and influencing factors in non-emergency medical transfers: a multiple linear regression analysis of a cross-sectional questionnaire study

**DOI:** 10.1186/s12913-019-4638-4

**Published:** 2019-11-07

**Authors:** Johann Georg Keunecke, Christine Gall, Torsten Birkholz, Andreas Moritz, Christian Eiche, Johannes Prottengeier

**Affiliations:** 1 Faculty of Medicine, Friedrich-Alexander University Erlangen-Nuremberg, Erlangen, Germany; 2 Department of Medical Informatics, Biometry and Epidemiology, Friedrich-Alexander University Erlangen-Nuremberg, Erlangen, Germany; 3 Department of Anaesthesiology, University Hospital Erlangen, Erlangen, Germany

**Keywords:** Emergency medical services, Human factors, Paramedics, Teamwork, Workload

## Abstract

**Background:**

Human workload is a key factor for system performance, but data on emergency medical services (EMS) are scarce. We investigated paramedics’ workload and the influencing factors for non-emergency medical transfers. These missions make up a major part of EMS activities in Germany and are growing steadily in number.

**Methods:**

Paramedics rated missions retrospectively through an online questionnaire. We used the NASA-Task Load Index (TLX) to quantify workload and asked about a variety of medical and procedural aspects for each mission. Teamwork was assessed by the Weller teamwork measurement tool (TMT). With a multiple linear regression model, we identified a set of factors leading to relevant increases or decreases in workload.

**Results:**

A total of 194 non-emergency missions were analysed. Global workload was rated low (Mean = 27/100). In summary, 42.8% of missions were rated with a TLX under 20/100. TLX subscales revealed low task demands but a very positive self-perception of performance (Mean = 15/100). Teamwork gained high ratings (Mean TMT = 5.8/7), and good teamwork led to decreases in workload. Aggression events originating from patients and bystanders occurred frequently (*n* = 25, 12.9%) and increased workload significantly. Other factors affecting workload were the patient’s body weight and the transfer of patients with transmittable pathogens.

**Conclusion:**

The workload during non-emergency medical transfers was low to very low, but performance perception was very positive, and no indicators of task underload were found. We identified several factors that led to workload increases. Future measures should attempt to better train paramedics for aggression incidents, to explore the usefulness of further technical aids in the transfer of obese patients and to reconsider standard operating procedures for missions with transmittable pathogens.

## Background

The number of missions carried out by Emergency Medical Services (EMS) has been growing steadily over the past years and is predicted to continue worldwide for the foreseeable future [1, 2]. Next, to the eponymous emergency missions, EMS in many countries must also carry out non-emergency medical transfers. Depending on healthcare system structures and EMS organizational make-up and location, this supposedly secondary activity may occupy large quantities of available EMS resources. Data published by the state of Bavaria show that 43% of all missions in the year 2017 were non-emergency missions [3]. In contrast to what their title might suggest, emergency paramedics may find themselves performing non-emergency duties on a regular basis [1, 3]. Considering the current demographic increase in immobile elderly patients and obese patients, as well as the ongoing concentration of hospital resources, it is conceivable that the number of non-emergency transfers will also increase substantially [4, 5]. As a result, the EMS work force assigned to conduct these transfers could also grow.

In contrast to the obvious importance and dynamics of non-emergency transfers, there is a lack of scientific data concerning the human factors behind the work in this subset of EMS activities. Such explorations of human factors and ergonomics aim to simultaneously improve system performance and paramedics’ well-being. Understanding the human factors in non-emergency transfers might help to increase efficiency and lower costs and thus be vital to resolve the modern health-care dilemma of simultaneous performance and cost pressure.

One of the core components and major contributors to both human performance and well-being is the workload associated with the tasks required [6, 7]. Workload is the psychological concept of subjective demands and self-perception, and a variety of definitions of workload do exist. For our study, we defined workload as the value on the NASA-Task Load Index scale. Yerkes and Dodson demonstrated that task overload, as well as task underload, will lead to a decrease in performance [8]. A medium workload level results in optimal performance [9].

Our study assessed the single-mission workload of non-emergency medical transfers conducted by EMS paramedics and aimed to identify relevant factors that influence workload. Our central objective was to detect these influencing factors. Using a network of relevant stakeholder organisations in pre-hospital emergency medicine, we conducted a national prospective survey to gather representative data. Workload was measured by the NASA Task Load Index (NASA-TLX), and we investigated a set of candidate variables that were assumed to possibly have an influence on workload, such as medical or organizational aspects of each transfer, paramedics’ teamwork, and their interactions with patients and third parties.

## Background information: German EMS

The German EMS is an emergency-physician-based system. While non-emergency transports and minor emergencies are autonomously managed by paramedics, emergency physicians are called to the scene for life-threatening emergencies.

Different levels of paramedical qualifications do exist in Germany. The “Rettungssanitäter” is trained in 13 weeks and has subordinate duties and responsibilities, while the “Notfallsanitäter” requires 3 years of training, which allows him or her to administer life-saving medication and to carry out life-saving measures while the physician is on his or her way to the scene. The previous qualification “Rettungsassistent” was replaced by the “Notfallsanitäter” in 2014. It required a two-year training that was similar but of lesser extent than the training of the “Notfallsanitäter”.

While BLS-ambulances (Basic Life Support) require at least one “Rettungssanitäter” on board, ALS-ambulances (Advanced Life Support) are staffed with at least one “Rettungsassistent” or “Notfallsanitäter”. Small variations in the crew lineup exist from state to state, as the EMS legislation is regulated on a state-level.

## Methods

### Study design

This national prospective observational study was designed as an online questionnaire survey. It took place over a period of 7 weeks in the autumn of 2017 and was approved beforehand by the research ethics committee of the Friedrich-Alexander-University Erlangen-Nuremberg under decision number 172_17B. Participation was anonymous, voluntary and unpaid. The study was promoted with information material sent out to all rescue stations nationwide as well as advertisements printed in all relevant German emergency medicine journals. The campaign was supported by major German EMS providers (Bayerisches Rotes Kreuz - Rettungsdienst, Arbeiter Samariter Bund Bayern - Notfallhilfe) as well as the paramedics’ labour union (Ver.di Bayern) and the paramedics’ professional society (Deutscher Berufsverband Rettungsdienst).

Paramedics scored non-emergency medical transfers retrospectively. A variety of candidate variables were considered as possible influencing factors, while workload was considered as the resulting parameter.

The compiling of candidate variables was achieved through structured discussions within a panel of human factor and emergency medicine experts from the University of Erlangen-Nuremberg. This committee consisted of three physicians (specialists in anaesthesia and emergency medicine) and two paramedics. All five were active medical simulations and team resource management trainers with longstanding experience in pre-hospital emergency medicine. In several meetings, each committee member had the equal right to bring variables to the discussion table.

### Medical and logistical mission aspects as possible influencing factors

Candidate variables included the patients’ general characteristics such as weight, their medical condition as classified by the NACA score, logistical consideration such as missions in overtime, procedural aspects such as the precautions against transmittable pathogens and interactions with others such as aggressive behaviour from patients and by-standers. While a set of variables was included in the final statistical model others failed in terms of statistical significance. Table 6 shows the variables included in the model, while Table 7 reports on the remaining.

### The quality of teamwork as a possible influencing factor

In addition to the above-mentioned candidate variables, we suspected teamwork to be a significant contributor to workload. To evaluate the quality of teamwork through self-assessment, the Weller teamwork measurement tool (TMT) was used. It is particularly well suited for use by professional medical emergency teams [10]. The original English version is known to have a valid structure for self-assessment [10]. For our purposes, we translated Weller’s TMT into German. Weller et al. defined 20 items to measure teamwork in emergency situations [11]. These 20 items are clustered in three main sectors: *leadership and team coordination* (LTC), *verbalizing situational information* (VSI), and *mutual performance monitoring* (MPM) [10]. For our study, we queried all 20 items to statistically evaluate not only the TMT score in total but also the means of each cluster (LTC, VSI, and MPM). This resulted in a score for teamwork ranging from 1 (low) to 7 (high). The same scale applied to the sub-scales of the TMT score.

### NASA task load index as an evaluation tool for subjective workload

The National Aeronautics and Space Administration Task Load Index (NASA-TLX) is a sensitive and validated measurement tool used to quantify subjective workload during a task or directly afterwards [6]. Originally designed to fulfil the needs of the aerospace industry, TLX is now widely used in different sectors, such as high-risk industries and medicine [12]. In the medical field, it has been used to measure workload in paediatric, sepsis and resuscitation scenarios [13, 14], during surgical procedures [15], in the emergency department, and in prehospital emergency medicine [16–18]. The NASA-TLX questionnaire is available in various languages [12]. As it is described that there are no significant differences between the results of paper-based and digital NASA-TLX questionnaires, we integrated the digital version into our study [19].

As measuring task load is subject to inter-individual variability, the Task Load Index’s goal was to minimize this spread in data [6]. Six sub-dimensions were identified to measure the task load with very low inter-individual variability: *physical, mental and temporal demands next to effort, performance,* and *frustration perception* [12]. All six sub-dimensions were included in our questionnaire, and the resulting global TLX was then calculated (Table 1). With its six subscales ranging from 1 to 100, the overall TLX is the arithmetic mean of all subscales again ranging from 1 to 100 [12]. However, understanding workload depends not only on the absolute values of the TLX but also on the relation of sub-dimensions to each other. The six sub-dimensions can be grouped into two separate causal mechanisms behind workload that need to be considered for their complex interdependence: While physical, mental and temporal dimensions represent how individuals perceive what the task demands from them, the (resulting) dimensions of effort, performance and frustration characterise how individuals perceive themselves and their actions in relation to the task.

**Table 1:** Wording of the NASA-TLX questionnaire

TLX-dimension	Wording (scale)
Mental Demand	How much mental and perceptual activity was required? Was the task easy or demanding, simple or complex? Was high precision required or was the task fault-tolerant? (1 = low, 100 = high)
Physical Demand	How much physical activity was required? Was the task easy or demanding, slack or strenuous? (1 = low, 100 = high)
Temporal Demand	How much time pressure did you feel due to the pace at which the tasks or task elements occurred? Was the pace slow or rapid? (1 = low, 100 = high)
Overall Performance	How successful were you in reaching your goals or the goals set by your team leader? How satisfied were you with your performance? (1 = good, 100 = bad)
Effort	How hard did you have to work to accomplish your level of performance? (1 = low, 100 = high)
Frustration	How irritated, stressed, and annoyed versus content, relaxed, and complacent did you feel during the task? (1 = low, 100 = high)

This table represents the NASA TLX questionnaire that was used to measure workload in our study. For each question, a slide bar allowed the participant to rate his subjective perception of workload from 1 to 100

### Data collection and statistics

For questionnaire access, the online platform SoSci Survey [20] was utilized. The data we gathered are available via a persistent Digital Object Identifier (DOI) linking to the dataset stored in the Zenodo data repository. Statistical analysis was performed using SPSS Statistics 24.0.0.0 (IBM Corp. Armonk, NY, USA), and statistical significance was defined as *p* < 0.05. Values are presented as the means with standard deviations and medians with interquartile ranges, where appropriate. The literature is inconsistent with regard to describing the statistical measures of NASA-TLX values. Both mean/SD and median/IQR reporting can be found. To allow for better comparison with previous data, we reported the NASA-TLX in both manners.

The global TLX was defined as our primary parameter of interest. To explore the influence of candidate variables on the TLX, we carried out a multiple linear regression analysis with a stepwise selection of predictor variables. Workload, defined as the value on the NASA-TLX scale, was the dependent variable, whereas mission characteristics and others were used as the independent variables.

For methodological clarity, we restricted the candidate variables to factors originating from the individual missions. In a separate investigation, our group recently studied the possible influences of professional biography, long-term characteristics and persuasions of paramedics’ on job-perception including perceived long-term workload [21].

Paramedics were asked to score both emergency missions and non-emergency medical transfers after each task. This article focuses on non-emergency transfers only. Our findings on workload and contributing factors from emergency missions are reported elsewhere [22].

## Results

### Study characteristics

A total of 98 participants recorded data on 260 non-emergency medical transfers. Each participant entered between 1 and 15 non-emergency missions (Median = 1; IQR = 1–2). A total of 194 mission documentations had complete NASA Task Load Index values and could thus be included for analysis. Table 2 shows the descriptive statistics of the participants:

**Table 2:** Descriptive statistics of the participants

		Min	Max	Mean	SD	n (%)
Sociodemographic data	Age	19	55	34.89	10.63	–
	Body Mass Index	19.57	44.31	28.00	5.91	–
	Years in job	0	37	12.53	10.24	–
Sex	Male	–	–	–	–	57 (58.17%)
	Female	–	–	–	–	18 (18.37%)
	Missing	–	–	–	–	23 (23.47%)
Qualification	Notfallsanitäter	–	–	–	–	30 (30.61%)
	Rettungsassistent	–	–	–	–	24 (24.49%)
	Rettungssanitäter	–	–	–	–	15 (15.31%)
	Notfallsanitäter in training	–	–	–	–	5 (5.10%)
	Other or not specified	–	–	–	–	24 (25.49%)

**Fig. 1:**
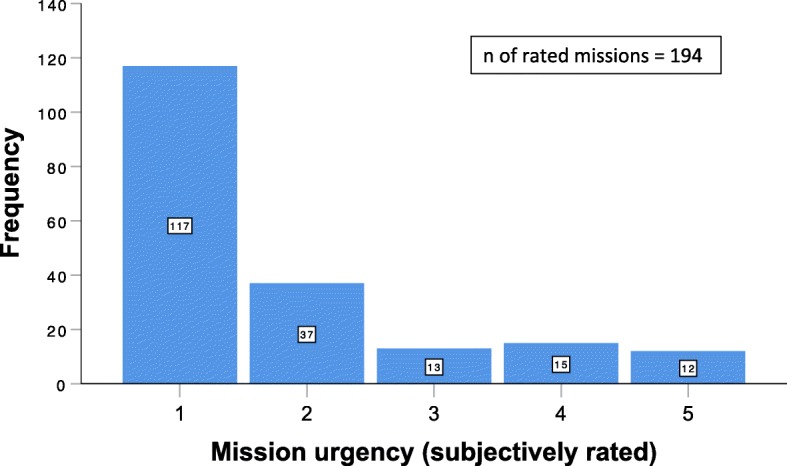
Subjectively rated mission urgency. The figure shows how the subjectively felt urgency of the single mission was perceived. The urgency was measured on a self-designed 5-step scale

The subjectively felt urgency of transfers was measured on a self-designed 5-point scale: 1 indicated no urgency and 5 indicated the highest urgency. Figure 1 shows the statistics of the subjectively felt urgency.

Of the 194 transported patients, 13 were rated as carrying transmittable pathogens (6.7%). Three cases (1.5%) were reported in which bystanders obstructed the patient’s treatment. Physically aggressive patients appeared in four missions (2.1%), whereas seven cases (3.6%) of verbally aggressive bystanders were documented. In total, 25 transfers (12.9%) were affected by aggression incidents (characterized as physically or verbally aggressive patients or bystanders or obstruction of treatment). More detailed descriptive statistics on aggression incidents (multiple answers possible) are shown in Table 3:

**Table 3:** Descriptive statistics of aggression incidents

	*n* (% of total missions)	Signs of intoxication (alcohol or drugs) *n* (%)
Physically aggressive patient	4 (2.1%)	2 (50.0%)
Verbally aggressive patient	20 (10.4%)	8 (40.0%)
Resistance to treatment	10 (5.2%)	2 (20%)
Physically aggressive bystander	0 (0.0%)	0 (0.0%)
Verbally aggressive bystander	7 (3.6%)	2 (28.6%)
Obstruction by bystander	3 (1.5%)	1 (33.3%)

This table gives an overview of the frequency of aggression incidents. Absolute numbers and relative frequencies are shown. Total n of mission = 194

### Weller teamwork measurement tool (TMT)

**Fig. 2:**
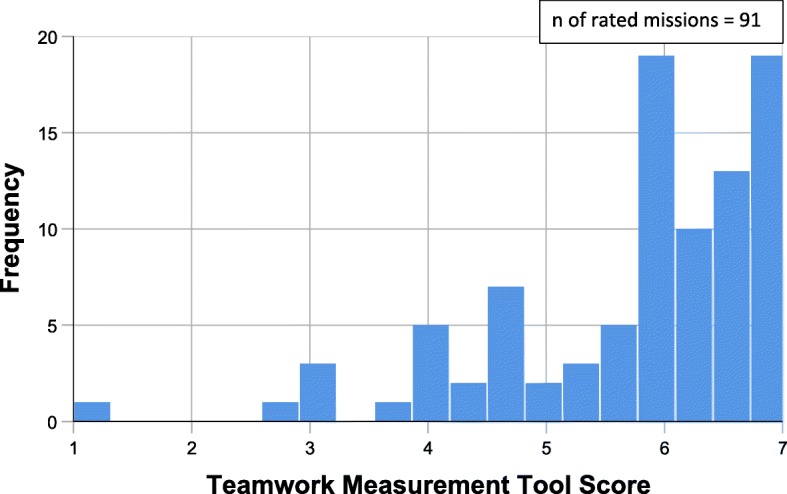
Distribution of the Teamwork Measurement Tool Score (TMT-Score). The figure shows how Teamwork was rated based on a single-mission rating using the Weller Teamwork Measurement Tool. Total n of non-emergency medical transfers in this study = 194

On Weller’s TMT scale (ranging from 1 to 7), teamwork averaged 6.28 (SD 0.93). Table 4 provides details of the descriptive statistics for the TMT, and Fig. 2 provides detailed information on the distribution of the TMT Score.

**Table 4:** Descriptive statistics of Weller TMT (Teamwork Measurement Tool) and its subscales

	Mean (SD)	Median (IQR)
Leadership and team coordination	6.28 (0.93)	6.57 (6.00–7.00)
Verbalizing situational information	5.23 (1.60)	5.59 (4.00–6.71)
Mutual performance monitoring	5.88 (1.60)	6.58 (5.50–7.00)
Overall TMT Score	5.77 (1.19)	6.02 (5.17–6.67)

The table shows the average values of the TMT and its three subdimensions

**Fig. 3:**
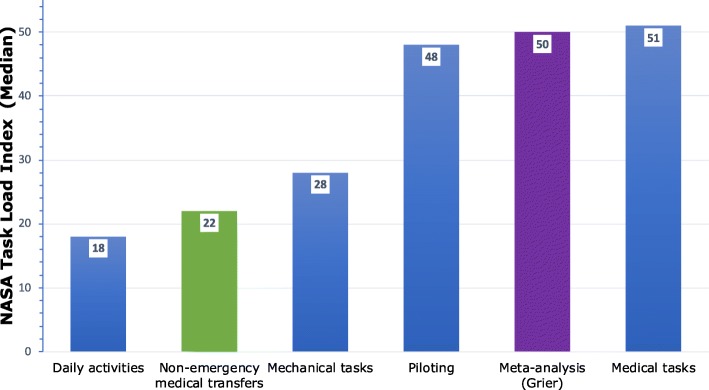
Comparison of median NASA-TLX values. The figure shows a comparison of the median TLX values between different activities and tasks. Data on tasks other than non-emergency medical transfers were reported previously [23]

### NASA task load index

The mean task load measured using the National Aeronautics and Space Administration Task Load Index (NASA-TLX) was 27 (SD 18). While 83 missions (42.8%) had a global TLX below 20, only 2 missions scored above TLX 80. Table 5 provides details of the descriptive statistics for the six subscales and the overall score of the NASA Task Load Index.

**Table 5:** Descriptive statistics of NASA-TLX and its sub-dimensions

	Mean (SD)	Median (IQR)
Mental demand	25 (29)	12 (2–46)
Physical demand	34 (28)	26 (10–56)
Temporal demand	18 (24)	5 (1–23)
Performance	15 (19)	8 (1–21)
Effort	30 (27)	21 (5–50)
Frustration	39 (35)	28 (5–72)
Global TLX	27 (18)	22 (13–38)

This table shows the average values of the NASA TLX and its six subscales. For a better comparison with existing literature, the mean and median are reported

## Multiple linear regression model

A multiple linear regression model was computed to investigate predictor variables that had a significant influence on the global NASA-TLX.

The subjectively felt urgency of transports was rated on a scale between 1 (no urgency) and 5 (highest urgency) by paramedics. There was a 6.9-point increase in the NASA-TLX for each positive step on the urgency scale (*p* < 0.01). Transporting a patient with potentially transmittable pathogens increased the task load by 15.4 points (*p* < 0.01). An increase of 0.1 points was caused by each additional kilogram of the patient’s body weight (*p* = 0.012). In cases where treatment was obstructed by bystanders, the task load increased by 35.0 points (*p* = 0.013). Verbal aggression from bystanders (delta 19.4 points, *p* < 0.01) or physically aggressive patients (delta 33.6 points, *p* < 0.01) also led to major increases in workload.

Each positive step on the 7-step teamwork measurement tools (*p* < 0.01) brought about a decrease in task load of − 4.0 points.

Details of the multiple linear regression model can be found in Table 6.

**Table 6:** Results of the stepwise multiple linear regression model

	Unstandardized Coefficients	Std. Error	Standardized Coefficients	Sig.	95.0% Confidence Interval for B	
	*B*		Beta		Lower Bound	Upper Bound
Constant	− 76.71	22.03		0.001	− 120,567	− 32.857
Urgency	6.87	1.19	0,475	0.000	4.495	9.235
Weller TMT	−3.96	1.33	−0.242	0.004	−6.606	−1.32
Physically aggressive patient	33.56	9.60	0.261	0.001	14.449	52.679
Infectious patient	15.42	4.86	0.243	0.002	5.733	25.096
Obstruction by bystander	35.05	13.82	0.194	0.013	7.536	62.553
Verbally aggressive bystander	19.43	7.06	0.211	0.007	5.372	33.488
Patient’s body weight	0.14	0.06	0.193	0.012	0.033	0.251

This table reports the results of our main statistical analysis. The total n of analysed missions was 194. Unstandardized coefficients explain how much the NASA-TLX value increases for one step on the scale of the variable that is shown in the first row. (F (7;79) = 15,018; *p* < 0.01; adjusted R2 = 53%; SE = 13%)

During the process of stepwise integration of predictor variables, some failed in terms of statistical significance. Table 7 lists variables that had been selected to undergo investigation but had to be removed from the multiple linear regression model because of low significance or low correlation with the TLX.

**Table 7:** Variables excluded from the stepwise multiple regression analysis

Scales	Incidents	Conditions
Indication	Verbally aggressive patient	Mission caused overtime
NACA score	Resistance to treatment	Missing equipment
	Intoxicated patient	Missions outside own precinct
	Accusation of having made a mistake	Paediatric mission
	Accusation of being late	Psychiatric patient
	Intoxicated bystander	Relatives present at the scene

Some variables that our committee thought had an influence on the NASA-TLX had to be removed from the analysis due to low significance or low correlation with the TLX. These excluded variables are shown in the table above

## Discussion

### Workload and performance

Workload is a key component of human performance. In this study, we quantified the workload for 194 non-emergency medical transfers conducted by EMS paramedics. Multiple linear regression analysis identified relevant factors influencing workload levels.

**Fig. 4:**
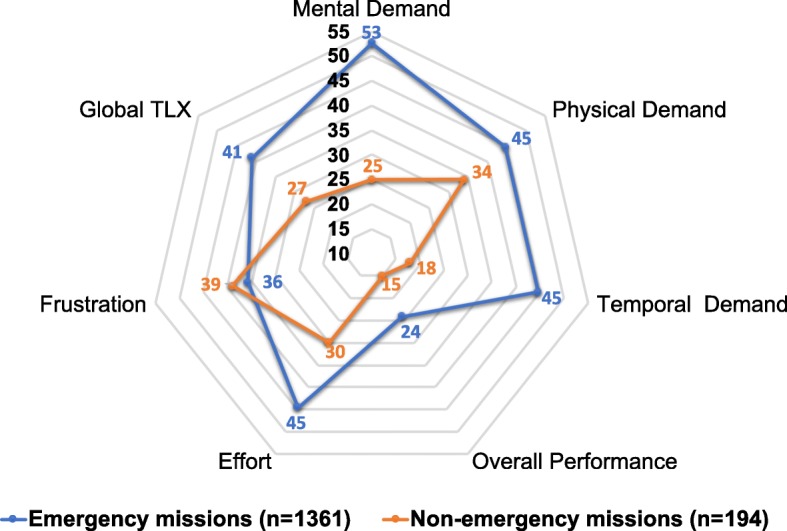
Comparison of the NASA-TLX subscale between emergency missions and non-emergency medical transfers. The figure shows a comparison of NASA-TLX subscale means between emergency missions and non-emergency medical transfers as rated by the participating paramedics. Data for the TLX subscales of the emergency missions were taken from a previous study [22]

The average overall workload in our data set as described by the global NASA-TLX was low compared to most other professions and tasks from previous studies. In a comprehensive 2015 meta-analysis, Grier calculated the median TLX-score for more than 1100 observations and 20 different task types at a median of 50 points [23]. In comparison, the median TLX of 22 for non-emergency transfers from our study ranged significantly lower than scores from mechanical tasks, piloting, or other medical tasks (Fig. 3). The workload from non-emergency EMS transfers was also significantly lower than the workload from EMS emergency missions (Fig. 4) that we analysed in a recent study [22]. In fact, the only tasks scoring somewhat lower in the Grier meta-analysis were so-called “daily activities” [23, 24]).

Eighty-three transfers (42,8%) were scored with a global workload below 20, reaching the bottom fifth of the NASA-TLX scale. Task Load Index sub-dimension analysis revealed that demands were unequal contributors to overall workload, as Table 4 shows. An interesting finding was that according to their performance self-ratings, paramedics usually felt satisfied and successful in reaching the goals of their tasks.

The frequent findings of low to very low TLX ratings must be weighed carefully. Yes, there is a broad consensus that workload and performance output are linked by an inversely U-shaped correlation [25]. However, several limitations apply. First, science has not been able to define a fixed TLX cut-off value that truly characterizes workload as being “too low” or “too high”, despite decades of research. This lack of a clear red line may be caused by inter-individually varying coping strategies, knowledge, and experience of persons undertaking the tasks on the outskirts of the workload continuum [23, 24]. Second, the association of workload and performance is much less understood for the lower end of workload than for upper extremes. With rising demands, efforts from workers will increase, but once the requirements exceed their capabilities, a degradation of performance output will follow with clear predictability. For example, a study in the context of emergency medicine demonstrated a positive correlation between workload excess in emergency departments and unwanted incidents negatively impacting patient safety [26].

On the other end of the scale, low demand tasks (meaning low physical, mental and temporal demands) do not automatically lead to poor performance output. Emotional attributes can pivot the individuals’ experience. Even in a low-demand-task, output can be reliably high if individuals have a strong feeling of successfully reaching their goals against a weak feeling of frustration and stress. Moreover, from our context, non-emergency transfers may be low-demand tasks, but they are very satisfying and performance output is perceived as being very high. Unfortunately, linear regression modelling identified several distinct factors in our data set that impact workload significantly and could therefore endanger this balance and lead to output deficits.

### Aggression incidents and workload

We recorded aggressive behaviour in words and actions by both patients and bystanders at a troublesome frequency. The variables in our model describing these incidents are the following: “physically aggressive patient”, “verbally aggressive bystanders”, and “obstruction by bystander”. All three events were linked to great increases in global workload. Other variables recording similar behaviour were not integrated into our final model due to missing statistical significance and can therefore be found in Table 7. Confounding with other aggression events may be responsible for this statistical conclusion.

Our findings are consistent with previous studies reporting increases in aggressive behaviour of patients worldwide. Gillespie reported that between 51 and 67% of American emergency department workers faced physical violence and 78–83% encountered verbal aggression during a study period of 18 months [27]. Authorities in the German state of North Rhine-Westphalia investigated violence against paramedics and found that within 12 months, 59% of personnel experienced physical aggression and 98% experienced verbal attacks [28].

Previous research has identified risk factors and early warning signs that help to predict aggression incidents: hostile behaviour, recent drug or alcohol misuse, non-adherence to psychotherapy or medication, and poor impulse control [29, 30]. Our data support these findings, as verbally or physically aggressive patients were often described as intoxicated in our cohort. As a consequence, the training of paramedics to identify these early indicators of aggressive incidents as well as de-escalation management could be implemented into paramedic education as these measures have proven themselves effective in other healthcare settings [31].

### Obesity and workload

The increasing prevalence of obesity worldwide has resulted in a rising incidence of overweight patients within healthcare systems and in an increasing number of ambulance transfers of overweight patients [5, 32]. Our data show that patients’ body weight leads to a significant increase in paramedics’ workload per kilogram of weight. It has long been known that lifting and transferring obese patients is difficult ergonomically, and a variety of presumably helpful technical devices have been introduced into daily EMS routine to manage the problem [33].

It is somewhat discouraging that even in our selected group of non-emergencies, when time should allow for proper preparation of lifting and transfer, the patient’s body weight still had such a profound effect on workload despite all aids and preventative measures. Therefore, the development, acquisition, and distribution of helping aids as well as operational procedures for the transfer of obese patients should be reconsidered. For example, a more liberal provision and dispatch of dedicated “heavy lift” ambulances could provide relief to regular ambulance crews and should be investigated for possible benefits on workload [33].

### Transmittable pathogens and workload

The prevalence of pathogens with antimicrobial resistance is rising in healthcare systems around the globe [34]. The transfer of patients with these and other transmittable pathogens have increased accordingly. The variable “infectious patient” specifies these missions. In German EMS, standard operational procedures (SOP) to prevent the transmission of these pathogens are long-established and comprise protective clothing, contact restrictions and subsequent cleaning of ambulances, among measures. The common basis of these SOPs is the “TRBA 250” – German nationwide technical rules for biological materials [35]. Although these SOPs are well-rehearsed and non-emergency transfers should provide ample time for preparation and implementation, such cases still lead to major increases in workload (plus 15 points).

It should be unfortunate that scenarios for which we supposedly already had good practice recommendations in place still have such a significant impact on workload. As with the obesity problem described previously, our data again necessitate re-consideration of SOPs, technical aids, and training for their benefit in these scenarios. Nassauer’s statement that intensified education is needed to avoid needless measures and reduce deficits in the knowledge about handling infectious patients is consistent with this goal [36].

### Teamwork and workload

In non-emergency transfers, different patients, different referring or receiving institutions, and frequently changing team partners can lead to challenging external conditions for teamwork. The connection of teamwork and performance output is considered of high value in ergonomics and over the last decades has led to “a golden age of interest in team research” [37]. In our cohort of non-emergency transfers, the subjective rating of teamwork was very positive across all sectors of the Weller TMT, and *leadership and team coordination* scored excellent values. We interpret these findings as the well-deserved payoff from the intensive efforts within German EMS to foster crew resource management as a core component of paramedic training. Good teamwork is not the only goal, as our results demonstrate: good teamwork leads to significant decreases in workload!

### Non-emergency transfers and medical conditions

In a recent study of paramedics’ workload from emergency missions, we identified medical aspects of missions such as the patient’s NACA score, scenarios such as multiple trauma, and the need for advanced procedures like airway management as factors to increase workload [22]. In comparison, in our cohort of non-emergency transfers, NACA scoring was significantly lower, emergency scenarios were not present, and advanced procedures were extremely rare occurrences. As a logical consequence, none of these candidate variables was selected for the workload model. However, the perceived urgency of a transfer did increase workload significantly and may serve as a surrogate parameter for the overall severity of a patient’s medical condition.

### Limitations

Our study had several limitations. The study was widely advertised and extensively distributed across German EMS, but we still cannot rule out a relevant selection bias in the participants or in the documented transfers. A methodological weakness of studies like ours is that the data may be biased by the healthy worker effect [38] or by an unwanted special appeal to paramedics with either very high or very low motivation for their job and for non-paid, voluntary scientific studies.

Second, our data consisted of self-reported subjective perceptions only. These may be prone to hindsight errors or other psychological influences and were not coupled with objective, i.e., physiological, measurements of workload.

This limitation also includes the subjective ratings of performance levels. Performance in non-emergency medical transfers was not measured in our study, and the literature does not provide any guidance on how to measure performance in such a multi-dimensional task. To overcome these limitations, future studies should try to provide measurement tools to objectively evaluate all subdimensions of the workload as well as external performance. Such efforts might also help to more clearly define the long desired “red-line” of workload.

Third, the questionnaire uses some “self-designed” items that have not undergone formal validation studies. These items have been highlighted as such in the text.

Finally, our data are missing ICU transfers. These specific missions are an important part of EMS providers’ scope of service and are also growing in number [3]. It seems worthwhile to investigate workload in this subspecialty in the future. The combination of critically ill patients, advanced use of medical equipment and team building between ICU physicians and EMS paramedics presents a unique background for human factor research.

## Conclusion

Our prospective study provides novel data on the human factors at work in emergency medical services. In a self-assessment with the NASA Task Load Index, EMS paramedics performing non-emergency medical transfers rated workload as low. Scores were lower than for EMS emergency missions and lower than for typical medical tasks from previous studies. Nevertheless, there was no indication of task under-load, and paramedics shared a common perception of successful performance and accomplishment.

Through regression modelling from candidate variables, we were able to identify factors that significantly increase workload, such as aggression events, patient weight, and transfer of patients carrying transmittable pathogens. In addition to a general rating of the cases’ urgency, no other factors relating to the patients’ medical condition contributed significantly to the workload. Team performance between paramedics was rated as very positive, and such positive ratings led to significant workload decreases.

Compared to emergency missions, non-emergency transfers resulted in lower and more homogenous workload ratings. For EMS organisations covering both types of missions, our study can indicate a way to homogenize the workload for the individual paramedics. Composite rosters - comprising emergency and non-emergency duties alike - could possibly lead to a more balanced workload and thus help to improve system performance and work satisfaction at the same time.

Workload increments from the transfer of obese patients and patients who are carrying transmittable pathogens can still be found regularly despite all previous preventative efforts to optimize such procedures. The re-evaluation of current measures seems indicated. Aggression events are reported with worrisome frequency. They lead to major increases in workload. Prevention and coping strategies should be brought into the focus of paramedics’ training.

Ultimately, our study provides baseline data for repeated workload assessments in the future to test the efficacy of possible improvement measures. The study can be part of a comprehensive strategy to optimize working conditions in EMS in times of growing case numbers, cost pressure and skilled labour shortages.

## Data Availability

The datasets generated and analysed during the current study are available in the Zenodo data repository: 10.5281/zenodo.2532618

## Abbreviations

DOI: Digital Object Identifier
EMS: Emergency Medical Services
ICU: Intensive Care Unit
LTC: Leadership and team coordination
MPM: Mutual performance monitoring
NACA: National Advisory Committee for Aeronautics
NASA: National Aeronautics and Space Administration
TLX: Task Load Index
TMT: Teamwork Measurement Tool
VSI: Verbalizing situational information
